# The Perceived Stress Scale for Kids (PeSSKi): Initial development of a brief measure for children aged 7–11 years

**DOI:** 10.1002/smi.3174

**Published:** 2022-06-17

**Authors:** Christina Davis, Julie M. Turner‐Cobb

**Affiliations:** ^1^ Department of Psychology Bournemouth University Poole UK

**Keywords:** child health, construct validity, perceived stress, reliability, scale development, self‐report

## Abstract

Perceived stress, a known risk factor for poor health, has been extensively assessed in adult populations. Yet an equivalent assessment tool for measurement of global perceived stress in children is lacking. This study aimed to develop and provide initial validation of a scale to measure perceived stress in children aged 7–11 years. Using a two‐phase design, we conducted semi‐structured interviews with thirteen child‐parent dyads for development of items. In a sample of 123 children (age range 7–11 years, *M*age = 9 years 7 months, 54.5% male) we administered the resulting Perceived Stress Scale for Kids (PeSSKi). Exploratory factor analysis of the 10‐item PeSSKi yielded support for both a one‐factor and a two‐factor solution (negative, positive item wording). The PeSSKi was associated positively with the Penn‐State Worry Questionnaire for Children (*r* = 0.748, *p* < 0.001) and negatively with the Students' Life Satisfaction Scale (*r* = 0.381, *p* < 0.001) indicating strong convergent/divergent validity respectively. Girls showed higher scores on the PeSSKi than boys with no effects observed by age. Initial psychometrics suggest the PeSSKi provides a robust scale for assessment of perceived stress in children. Further validation is needed across different child populations, over time and with physical measures of stress and health outcomes.

## 1 INTRODUCTION

The importance of subjective assessment of perceived stress in relation to health has become increasingly documented and widely accepted in stress research. Perceived stress has been associated with numerous physical and mental health sequelae (Slavich, 2020), with effects exerted across immune, neural and endocrine systems (Glaser & Kiecolt‐Glaser, 2005; Piccolo et al., 2018), leading to increased vulnerability to poorer health (Epel et al., 2004). Much work has utilised the adult Perceived Stress Scale (PSS) (Cohen et al., 1983), a widely used, psychometrically sound and extensively cited instrument, for the assessment of self‐reported global stress due to overwhelming or uncontrollable events. The PSS‐14 item and abbreviated 4‐item versions (Cohen et al., 1983), and subsequently the PSS‐10 (Cohen & Williamson, 1988) were developed for ages 18 years upwards. Additionally, there exists limited although encouraging validation of the PSS‐10 in adolescent populations from age 12 years (Kechter et al., 2019; Liu et al., 2020). Whilst Cohen and Williamson (1988) identified a two‐factor structure for the original PSS‐14 and subsequent PSS‐10, due to positively and negatively worded items, they assert that an overall one‐factor score is the most pertinent to assessment of perceived stress and view “the distinction between the two factors …[as] irrelevant” (p.45) in this context. Debate ensues over this issue and more recent work has pursued the two‐factor solution arguing for the relevance of two subscales in relation to assessment of the PSS‐10 across a range of populations and cultures (e.g. Baik et al., 2019; Golden‐Kreutz et al., 2004; Wang et al., 2011; Örücü & Demir, 2008). Different labels assigned to these negative and positive subscales include ‘stress’ and ‘counterstress’ (Barbosa‐Leiker et al., 2013; Golden‐Kreutz et al., 2004), with the classification most frequently applied being ‘perceived helplessness’ and ‘perceived self‐efficacy’ (e.g. Liu et al., 2020; Roberti et al., 2006; Taylor, 2015) suggesting the distinction may serve theoretical underpinnings beyond item directionality (Baik et al., 2019).

Despite the importance of stress during childhood with implications for lifelong health outcomes (National Scientific Council on the Developing Child, 2014; United Nations, 2018), stress in children has frequently focussed on objective assessments or subjective measurement of specific events, rather than on perceived global stress. Several countries, consistent with recent global data, have reported an escalation in children experiencing significant mental distress (Abramson, 2022; Lennon, 2021; UNICEF, 2021) with intensity amplified due to the COVID‐19 pandemic (Jeffery et al., 2021). Stress during childhood is particularly salient, since it may augment brain development (Lupien et al., 2009) with potentially enduring effects (Pervanidou & Chrousos, 2018).

Child populations are limited in life experience and exposure (Ryan‐Wenger, 1992) compared to adolescent and adult populations, and concepts used in adult stress scales may not meaningfully translate to stress in children (Yamamoto et al., 1998). Adults also demonstrate poor ability to predict childhood stress (Amirkhan & Auyeung, 2007). Evidence suggests that child and adult responses to the same questions concerning a child's stress differ (Lyneham et al., 2013) and adult‐based assessment of children's lives may not accurately reveal the perspective of the child (Allwood et al., 2017). Furthermore, feelings of stress may develop gradually and go unacknowledged or become accepted as ‘normal’ by the child (Sieh et al., 2010). The process of physiological stress regulation requires a measure of emotional maturity (Klemfuss & Musser, 2020) that improves as part of normative development (Eisenberg et al., 2005). Ages 7–11 years constitute a critical phase in this process (Band & Weisz, 1988), since independent regulation is being developed through practice with significant adults (McRae et al., 2012). Additionally, sex differences in hypothalamic pituitary adrenal axis activity may be present in childhood (van der Voorn et al., 2017) with girls more reactive to acute stress than boys (Raffington et al., 2020). Evidence is inconsistent for chronic and perceived stress but suggests gender differences in stress profiles for children (Lindblad et al., 2008) and adolescents (Byrne et al., 2007). Such gender differences in health research point to a possible disparity in vulnerability to stress and subsequent disease pathways (Piccini et al., 2018).

Findings suggest that young children are not only familiar with but articulate in describing stress concepts (Band & Weisz, 1988; Cheetham et al., 2016; Cheetham‐Blake et al., 2019; Valentine et al., 2010) and that 7‐11‐years‐olds are competent in accurate self‐report (Bell, 2007) when questions asked are appropriately designed and worded (Borgers et al., 2004). Indeed, it is proposed that any study of child well‐being should incorporate the child's own evaluations as part of a multi‐disciplinary indicator (Ben‐Arieh, 2005). However, cognitive and emotional maturity may present difficulty in translation of emotional categories into more concrete concepts developmentally available for 7–11‐year‐olds (Zeman et al., 2007). Further confounds include linguistic limitations (Bell, 2007) and issues of social desirability (Miller et al., 2015). Evidence indicates that when presented with emotion‐based scales with negatively worded items, young children respond in polar extremes (Chambers & Johnston, 2002) since these require verbal reasoning at a higher level than positively worded items (Marsh, 1986). Young children may also struggle with time concepts when asked about their feelings or emotions (Zeman et al., 2007), yet many scales incorporate timeframes (Snoeren & Hoefnagels, 2014; White, 2014). Some child scales compromise brevity for depth producing lengthy assessments (Byrne et al., 2011; Maldonado et al., 2008) that increase concentration burden.

Consequently, assessment of perceived global stress in children is particularly poorly represented. We have identified three existing non‐proxy self‐report scales that specifically aim to assess perceived stress in children. Firstly, the 21‐item Stress in Children (SiC) questionnaire (Osika et al., 2007), designed for 9–12 year olds, provides a global score plus three factors (well‐being, distress, lack of social support), and avoids use of a timeframe response. However, the SiC scale includes somatic symptoms (e.g. *headaches*, *stomach pains*), a number of items about emotions (e.g. *I get angry* or *I get sad*) without contextualisation by stressor characteristic, and with reference to specific stressful events (e.g. being teased by other children) or specific domains of stress experience (e.g. school) (Osika et al., 2007). Secondly, the 19‐item Perceived Stress Questionnaire (PSQ8‐11) formulated for children aged 8–11 years with two subscales (psychological and physiological stress), includes somatic symptoms (e.g. *sweaty hands, feel nauseous*) and several health behaviour items relating to sleeping or appetite (Snoeren & Hoefnagels, 2014). The PSQ8‐11 uses a timeframe that asks children to report the frequency of experiences over the ‘last week’ (Snoeren & Hoefnagels, 2014). The third scale, the 13‐item Perceived Stress Scale for Children (PSS‐C) (White, 2014), uses a visual likert scale relevant to the younger child age group, but was designed for a wide age‐demographic (5–18 years), also employs a ‘last week’ frequency reporting timeframe and includes reference to school specific stressors and items relating to parental attachment (e.g. *… how often did your mom and/or dad make you feel loved?*). Rapid development in childhood represents a particularly heterogenous population and as such, scales designed for wide age‐groups fail to capture the particular linguistic and cognitive capabilities of each developmental stage. Whilst all three scales are comprehensive and show good internal reliability and validity, each has shortcomings that detract from being a comparable child equivalent to the PSS for adults. There exist more stress scales designed for adolescent age groups, such as the Chronic Stress Questionnaire for Children and Adolescents for 10–20 year olds (De Bruin et al., 2018) and the Adolescent Stress Questionnaire for 12–17 year olds (Byrne et al., 2007) but the language used and time concepts applied would not transfer easily to a younger demographic. Other available stress scales designed for younger children assess specific daily hassles (Byrne et al., 2011) or coping with stress (Gökler DanIşman et al., 2017) rather than global perceived stress.

It is evident that there exists a need and potential for development of an age‐appropriate self‐report measure of global perceived stress for younger children that addresses the issues identified in the small number of existing measures. The current study aimed to develop and conduct initial psychometric evaluation of a self‐report perceived stress scale designed for children aged 7–11 years; the Perceived Stress Scale for Kids (PeSSKi). We hypothesised that the PeSSKi would show a single factor structure and in addition aimed to explore other possible alternative solutions such as might be yielded by negatively and positively valenced wording of items. We also expected the PeSSKi to be positively associated with a measure of worry and negatively associated with a measure of life satisfaction, both established measures for use in children. Finally, we examined whether the PeSSKi would discriminate between genders and within the 7–11 age group.

## 2 METHODS

### 2.1 Participants and procedure

This study utilised an embedded design with initial development of the original item pool, prior to a two‐phase approach to data collection: (i) qualitative interviews for scale development and refinement; (ii) quantitative testing of the derived item set in an online questionnaire format. Ethical approval for both phases of the study was provided by the Science, Technology and Health Research Ethics Panel at the authors' institution (ethics ID #35415). For phase one interviews, 13 children aged between 7 and 11 years (8 males, 5 females) and one of their parents (mother in all cases) were recruited via personal invitation to parents, composing an opportunity sample of parents and children. Inclusion criteria were the ability to read and understand English, access to a computer or Internet device (for the purpose of Zoom interviews) and that the child was not currently undergoing treatment for an anxiety‐related mental health issue.

In phase two testing, participants were invited via direct email, social media adverts and email request to 14 schools from across the UK. This included two schools in close geographical proximity to the authors' University and the remainder selected at random from the UK government's Office for Standards in Education website (https://www.gov.uk/government/organisations/ofsted). Schools were asked to advertise the study in their newsletter; four schools responded and agreed to feature the advert. A total of 123 children aged 7–11 years were enroled (male = 67, female = 55, non‐binary = 1). Sample size was based on recommendations for best practice in scale development that suggest approximately 10 participants per newly generated scale item (Boateng et al., 2018) and considers greater than 100 participants as excellent for internal consistency (Aaronson et al., 2002). Factor analysis was considered warranted based on a ratio of 5–10 participants per item (Tinsley & Tinsley, 1987). A sample size of 100 participants plus approximately 25% to allow for any missing data was deemed sufficient.

#### 2.1.1 Initial item selection

The original item pool was generated through deductive and inductive processes. A literature search provided the theoretical basis for perceived stress and identified existing child scales. Items were underwritten by stressor characteristics such as feelings of control, ability to adapt to the unexpected, and feelings when exposed to uncertainty. Statements were carefully designed to be time‐ and context‐free, in order to capture general, global stress rather than being stressor specific. Item content was guided by findings from qualitative work assessing stress in children of the target age group (Cheetham‐Blake et al., 2019). Appropriate form, wording and response options for the scale were carefully considered through informal discussion with members of the target population and experts in the field of stress and child development. Content validity was examined and refined through evaluation of the items by academics and educators with expertise in child stress research. This generated an initial item pool of 20 statements (see Supporting Information S1, that lists the initial item pool and subsequent refinement of items), approximately double the length of the desired final scale (Boateng et al., 2018), necessitating discussion with the target population during interview.

#### 2.1.2 Phase one: Interviews

Semi‐structured interviews were conducted with 13 child‐parent dyads, based on a prepared interview schedule (see Supporting Information S1). Parent involvement was designed to enhance and facilitate discussion with the child and deemed to assist with enabling accurate memories and responses (Cheetham‐Blake et al., 2019) but the final item pool was driven by child, rather than parent responses. Due to restrictions created by the COVID‐19 pandemic, interviews were conducted over Zoom, recorded using a dictaphone and each lasted approximately 20–30 min. Written informed parental consent and child consent, together with a copy of the 20‐item question pool were distributed to the parents via email in advance of the interview. Child participants were also asked for verbal assent at the start of the interview. During interview, children were encouraged to talk about what they thought each item meant, give their opinions on whether they understood the item and provide suggestions on item improvement. At the end of each interview, parental opinion was sought separately from the child, allowing the parent to offer alternate input.

Interview data was stored anonymously on a secure database before being transcribed. Inductive analysis was performed for each interview to allow for item reduction ensuring the language, parsimony and internal consistency of the scale was correct. Interviews identified several words or concepts within the original 20‐item PeSSKi pool that were difficult to understand. These were removed or revised accordingly and in alignment with criteria for scale development (Streiner et al., 2015). Since the children were found to be more at ease with positive concepts and statements, we provided as many as feasible without interfering with the purpose of the scale. Additionally, the children preferred shorter statements with simpler linguistic frameworks and items that did not ask them to think about emotional concepts such as feeling scared, bad things happening or doing something wrong. Following reduction and selection of items, further informal expert opinion was sought from psychologists and school education providers. Literature reviews surrounding survey use with different groups were conducted to ensure the items were developmentally, theoretically, and linguistically appropriate for all members of the target population. The final PeSSKi for phase two testing consisted of 11 items.

#### 2.1.3 Phase two: Testing

A questionnaire was presented online via the survey hosting website, *Qualtrics*. It consisted of the PeSSKi scale plus two social desirability items, in addition to two comparison scales and was live from 31 March 2021 until 11 May 2021. To assist the children in their responses, and to acknowledge possible difficulties due to undisclosed dyslexia or sensory processing issues, certain elements of the design were included as recommended by the British Dyslexia Association (2018): background to the scale was off‐white; font colour a dark blue; and font size was large and evenly spaced without use of italics. A star‐response‐system was adopted with five stars indicating *a lot* (corresponding to five on a Likert‐scale), and 1 star indicating *not at all* (corresponding to one on a Likert Scale). Response option reminders were used, as well as child‐friendly pictures and encouragement at regular intervals to maximise completion and reduce fatigue.

### 2.2 Measures

#### 2.2.1 Demographic information

Parents provided initial details of the child's age (years/months), gender and household socio‐economic status (SES). Socio‐economic status was based on the highest self‐reported household occupation level using the UK Office for National Statistics (NOMIS) classification (https://www.nomisweb.co.uk) from 1 (managerial role) to 9 (not currently in paid employment).

#### 2.2.2 Psychosocial scales

Child participants provided responses to three scales relating to perceived stress, worry and life satisfaction, in addition to two social desirability items that were embedded within the PeSSKi scale.

## 3 PERCEIVED STRESS SCALE FOR KIDS (PeSSKi)

The preliminary PeSSKi scale consisted of 11 perceived stress statements and was presented with the insertion of two social desirability items, all measured on a 5‐point Likert scale ranging from 1 (not at all) to 5 (a lot) using a star response system (see Supporting Information S1, which shows the 11‐item PeSSKi with instructions and star response scoring). Perceived stress items included aspects such as feeling out of control of emotions or helplessness. Six items were positively valenced, and five were negatively valenced. For the 11 PeSSKi items scores were obtained by reversing the six responses to the positively worded items and then summing across all scale items. Summed scores had the potential to range from 11 to 55, where a higher number was indicative of a higher level of perceived stress. The PeSSKi was self‐report, with the opportunity for younger or less able participants to complete it under parental guidance if necessary. However, the scale was designed with the potential to be accessible to all ages within the 7–11‐year age group unaided. Reading ease for the PeSSKi was calculated using Flesch‐Kincaid grade within the Microsoft Word Readability Statistics tool. The two social desirability items (*I like everyone I have met* and *I always tell the truth*) were included for the purpose of scale development in this study and are not intended to be a permanent feature of the PeSSKi. These items were designed to reflect enhancement or denial as a function of social desirability (Paulhus & Reid, 1991) and were selected from the social desirability subscale of the Stirling Children's Well‐being Scale (SCWBS) (Liddle & Carter, 2015) in order to assess any possible influence of social desirability on responses to the PeSSKi scale items.

## 4 PENN‐STATE WORRY QUESTIONNAIRE FOR CHILDREN (PSWQ‐C)

The PSWQ‐C (Chorpita et al., 1997) is one of the most widely used instruments in assessing general characteristics of worry in children aged 7–17 years old. It aims to measure the propensity to participate in generalised and uncontrollable worry to a disproportionate degree (Muris et al., 2001) and is a modified version of the adult scale. The version used is reported as reliable (*α* = 0.89) and valid with other worry measures and forms a shortened 11‐item scale version of the original 14‐item scale (Muris et al., 2001). Measured on a 5‐point Likert scale ranging from 1 (*not at all*) to 5 (*a lot*) where a high score indicates a high state of generalised anxiety, a summed score was calculated. This scale was chosen to assess convergent validity given that considerable theoretical and empirical links have previously cited that the construct of worry is positively related to stress (van den Bergh et al., 2021). Cronbach's *α* for the PSWQ‐C in this sample was 0.94.

## 5 STUDENTS' LIFE SATISFACTION SCALE (SLSS)

The SLSS (Huebner, 1991) consists of nine self‐report items intended to determine children's global life satisfaction as a broad evaluation of their whole life rather than within specific domains and has been validated for young people aged between 8 and 18 years old. The scale is measured on a 5‐point Likert scale ranging from 1 (*not at all*) to 5 (*a lot*) with high scores indicating a high level of satisfaction with life (two negatively phrased items were reverse scored) and items summed. This scale has been utilised in a variety of settings including a reduced item version to assess children's perceived thoughts on life in the annual Good Childhood Report (e.g. The Children's Society, 2020). Internal consistency of the 9‐item scale has been reported as strong with an *α* of 0.84 (Huebner, 1991) and an *α* of 0.82 for the 7‐item version. In the current study we used the original 10‐item SLSS (Huebner, 1991) that included the item ‘I am happy with my life’, originally excluded by (Huebner, 1991) for semantic rather than reliability reasons. This corresponds with the single‐item measure of adult global happiness used in the UK Office for NOMIS, Measuring National Well‐being Programme (ONS, 2018). Cronbach's *α* for the 10‐item SLSS in the current sample was 0.88 (*α* = 0.87 for the 9‐item version in this sample).

### 5.1 Statistical analysis

Analysis for phase two testing was conducted using IBM SPSS Statistics (Version 27) with the dataset of 123 participants. Missing values were observed from three participants only, all of which were in the last items of the SLSS; these were deleted pairwise for analyses that included the SLSS. Analyses that involved gender as a variable included participants identifying as male or female (*n* = 122). Reliability coefficients (Cronbach's *α*) were initially examined for internal consistency on each of the scales in the sample using criteria (Boateng et al., 2018) where the minimum alpha for new scales should be 0.7. Items showing low correlation values with the overall score were considered for removal where supported by findings from the factor analysis. To assess any undue influence of social desirability on scale scores, correlation analyses were examined between each of the three overall scale scores and the social desirability score. Exploratory factor analysis was applied to the 11‐item PeSSKi using principal components analysis (PCA) extraction to explore factor structure. Suitability of the data for PCA was examined on the basis of coefficients reaching 0.3 and above, a Kaiser‐Meyer‐Olkin value of 0.6 and above and Barlett's Test of Sphericity achieving significance. The Monte Carlo PCA (Watkins, 2000) was used for Parallel Analysis to assist in identifying the number of factors for extraction. The structure matrix identified that item #10 did not correspond well with the other items and subsequent factor analysis was based on the 10‐item PeSSKi, excluding item #10. Exploratory factor analysis was conducted in the same manner using these 10‐items of the PeSSKi, with two follow up PCAs one using a one‐component and the other a two‐component fixed number of extractions. The 10‐item PeSSKi had a potential summed total score ranging from 10 to 50.

Evidence for divergent validity of the scale was assessed through examination of correlation coefficients (Pearson) between the PeSSKi and the SLSS. Divergent validity was considered appropriately discriminated when a correlation of less than 0.8 or a negative correlation was observed between constructs (Boateng et al., 2018). Convergent validity of the scale was assessed through correlation coefficients (Pearson) between the PeSSKi and the PSWQ‐C. Correlation coefficients between the PeSSKi and the comparison scales were assessed using partial correlations controlling for social desirability; correlation coefficients with and without social desirability were compared to assess its level of influence on the relationship between the two scores. Two‐way ANOVAs were conducted to examine effects of gender and age on each of the PeSSKi scores (two‐tailed effects reported). For this analysis the sample was divided into five equal groups by age (months). Tests of validity were conducted using the total PeSSKi score and the two PeSSKi factors suggested in exploratory factor analysis. Data on which these analyses were conducted are openly available at https://doi.org/10.18746/bmth.data.00000209.

## 6 RESULTS

### 6.1 Phase two: Scale testing

#### 6.1.1 Acceptability, accessibility and reading ease

All respondents completed the PeSSKi without omitting any items, indicating good acceptability. Reading ease was at Flesch‐Kincaid grade level 1.66, demonstrating accessibility of the scale to 7–9‐year‐old children. Anecdotal parental feedback reflected that participation ‘was a great way to understand more about how [their child] was feeling’, suggesting additional acceptability by parents.

#### 6.1.2 Reliability and inter‐item correlation

Internal consistency for the 11‐item PeSSKi was acceptable (Cronbach's *α* = 0.76) with the mean summed score of 27.93 (SD = 7.69) and range of 13–53. However, the corrected item‐total correlation of the PeSSKi revealed one item (#10 ‘There is always someone to talk to about what is bothering me’) with a value below the recommended 0.30, indicating this item to be less reliable as a measure of perceived stress in our sample (see Supporting Information S1). The inter‐item correlation matrix (see Table 1) corroborated this anomaly with negative correlation coefficients between this item and items #2 (‘I feel panicky or afraid about little things’) and #3 (‘I feel calm and relaxed’). The Cronbach's *α* after removing item #10 increased to 0.78 (mean summed score = 26.34, SD = 7.57, range = 12–50) suggesting that the scale is a better fit for the domain of perceived stress without the inclusion of this item. Exploratory factor analysis using the 11‐items provided further evidence that item #10 did not correspond well with the other items as indicated by unrotated and rotated factor loadings. Consequently, subsequent analyses reported are for the 10‐item PeSSKi with item #10 removed.

**TABLE 1:** Descriptive statistics and inter‐item correlations for the 11‐item PeSSKi (*N* = 123)

Item #	For each sentence, please show us in ⭐‘stars’⭐ how it best describes *you*	*M*	*SD*	1	2	3	4	5	6	7	8	9	10	11
1	I can think clearly (R)	1.97	1.01	‐										
2	I feel panicky or afraid about little things	2.62	1.44	0.32	‐									
3	I feel calm and relaxed (R)	2.41	1.28	0.48	0.27	‐								
4	I feel helpless when I have a problem	2.33	1.39	0.07	0.27	0.09	‐							
5	I feel fine with new things, new people, or new places (R)	2.55	1.39	0.28	0.20	0.22	0.16	‐						
6	I worry when something unexpected happens	3.07	1.28	0.17	0.37	0.28	0.28	0.34	‐					
7	I sleep well (R)	2.31	1.42	0.57	0.26	0.54	0.15	0.20	0.19	‐				
8	I get upset or angry easily	3.20	1.36	0.15	0.38	0.20	0.19	0.02	0.13	0.24	‐			
9	It is hard to calm down when my feelings are too big or too strong	3.20	1.36	0.09	0.36	0.14	0.31	0.17	0.30	0.23	0.51	‐		
10	There is always someone to talk to about what is bothering me (R)	1.59	0.922	0.00	**−0.18**	**−0.03**	0.16	0.09	0.00	0.09	0.13	0.01	‐	
11	Whatever happens I can cope with it (R)	2.69	1.81	0.29	0.17	0.30	0.26	0.41	0.24	0.30	0.37	0.23	0.16	‐

*Note*: Reverse scored items are indicated as (R). Negative correlation coefficients are indicated in bold.

#### 6.1.3 Validation checks using social desirability items

There was no significant correlation observed between the 10‐item PeSSKi total score and social desirability (*r* = −0.142, *p* = 0.117) or by gender (males, *r* = −0.209, *p* = 0.090; females, *r* = −0.048 *p* = 0.730). Assessment of the two PeSSKi factors (positively and negatively valenced items) with social desirability revealed no significant correlations for boys on either factor but for girls we found significant correlations for the negatively valenced PeSSKi factor (*r* = 0.281, *p* = 0.038) and for the positively valenced PeSSKi factor (*r* = −0.344, *p* = 0.010). This suggests that whilst the PeSSKi was overall not substantially influenced by social desirability, within the child sample tested there was some evidence that girls were significantly influenced by social desirability compared to boys, particularly for the positively valenced factor. There was no significant correlation between the PSWQ‐C and social desirability (*r* = −0.073, *p* = 0.421) and no significant effects by gender. A weak correlation was noted between the SLSS and social desirability (*r* = 0.210, *p* = 0.021) with a higher correlation for boys (*r* = 0.239, *p* = 0.053) than girls (*r* = 0.185, *p* = ,184) suggesting modest influence of socially motivated responses for global life satisfaction scores with differences by gender.

#### 6.1.4 Exploratory factor analysis

Suitability of the data for PCA was confirmed on the basis that several coefficients reached 0.3 and above, a Kaiser‐Meyer‐Olkin value of 0.74 was obtained and Barlett's Test of Sphericity achieved significance (*p* < 0.001). Three components were identified with eigenvalues above 1, explaining 33.67%, 14.58%, and 11.25% of the variance. Visual inspection of the screeplot indicated a definitive break after component 1 and a further point of decline after component 3, suggesting a one factor solution with a possible two factor solution worthy of exploration. Results of a Parallel Analysis (10 variables, 123 participants, 100 replications) confirmed this, yielding an eigenvalue for the first component considerably greater than the random generated eigenvalue and marginally greater for the second eigenvalue, with all other eigenvalues smaller than those randomly generated. Based on these findings, two PCAs were then conducted using a one‐component and a two‐component fixed number of extractions.

The one‐component solution explained 33.67% of the variance and the two‐component solution added a further 14.58% with a total cumulative variance of 48.26%. The one‐component extraction identified three items (#4 ‘I feel helpless when I have a problem’, #5 ‘I feel fine with new thing, new people, new places’, #8 ‘I get upset or angry easily’) in which the variance explained showed values less than 0.3, indicating these items as the weakest fit within the scale (see Supporting Information S1). In the two‐component extraction, the component matrix (unrotated items) showed all items to load most strongly on component one. A modest negative correlation was found between the two components (*r* = −0.355). Using oblimin rotation to interpret the two‐component structure, the pattern matrix indicated clear loading of items on either one of the two components, with item #11 (whatever happens, I can cope with it) showing correlation with both components and higher loading of this item on component 2. In this two‐component solution, only #5 (‘I feel fine with new things, new people, or new places’) was identified as explaining the least variance (0.267) of all items, suggesting this item as a less suitable fit with the other component items. Item loadings on the two components is consistent with the interpretation of negative valenced items (component 1) and positively valenced items (component 2) (see Table 2). Findings suggest that the total scale score can be used to assess overall perceived stress and further support the calculation of negative and positive subscale scores.

**TABLE 2:** Pattern and structure matrix for principal components analysis (PCA) with oblimin rotation of two factor solution with 10 PeSSKi items (*N* = 123)

PeSSKi item	Pattern coefficients		Structure coefficients		Communalities
	Component 1	Component 2	Component 1	Component 2	
9. It is hard to calm down …	**0.820**	0.124	**0.776**	−0.167	0.616
8. I get upset or angry easily	**0.696**	0.022	**0.688**	−0.225	0.474
4. I feel helpless when I have a problem	**0.643**	0.092	**0.610**	−0.136	0.379
2. I feel panicky or afraid about little things	**0.571**	−0.196	**0.641**	−0.399	0.444
6. I worry when something unexpected happens	**0.489**	−0.197	**0.559**	−0.371	0.347
1. I can think clearly	−0.129	−**0.861**	0.177	−**0.815**	0.679
3. I feel calm and relaxed	−0.042	−**0.798**	0.242	**−0.784**	0.616
7. I sleep well	0.006	**−0.788**	0.286	−**0.791**	0.625
5. I feel fine with new things, …	0.186	**−0.421**	0.335	**−0.487**	0.267
11. Whatever happens I can cope with it	0.363	**−0.385**	0.499	**−0.513**	0.379

*Note*: Bolded values indicate major loadings for each item.

#### 6.1.5 Construct validity

The 10‐item PeSSKi and the negatively and positive valenced factors were all significantly positively correlated with the conceptually analogous measure of worry (PSWQ‐C) (*r* = 0.748, 0.680, 0.584 respectively, all *p* < 0.001) indicating satisfactory convergent validity for the overall scale and two factors. Partial correlations between these PeSSKi variables (overall scale score, negative and positively valenced factors) and the PSWQ‐C controlling for social desirability revealed a negligible influence on the strength of these relationships (*r* = 0.747, 0.684, 0.589 respectively, all *p* < 0.001). Discriminant validity was demonstrated by a significant negative correlation between overall, negative and positive scores on the SLSS (*r* = −0.381, −0.222, −0.427 respectively, all *p* < 0.001). Controlling for social desirability had minimal effect on the outcome (*r* = −0.363, −0.234, −0.393, all *p* < 0.001).

#### 6.1.6 Criterion‐related validity

There was no significant interaction effect of gender and age on PeSSKi scores (overall, negative or positive factors). A significant main effect for the overall PeSSKi score was observed for gender, *F* (1, 112) = 7.90, *p* = 0.006, with a medium effect size (partial *η* ^2^ = 0.07); scores on the PeSSKi were found to be higher for girls (*M* = 28.27, *SD* = 6.90) than boys (*M* = 24.58, *SD* = 7.65). For the two PeSSKi factors, there was no significant effect for the negatively valenced factor but a main effect of gender for the positively valenced factor *F* (1, 112) = 9.68, *p* = 0.002, with a medium effect size (partial *η* ^2^ = 0.08); scores on the positively valenced PeSSKi factor were higher for girls (*M* = 13.13, *SD* = 4.52) than boys (*M* = 10.90, *SD* = 4.01). Compared to boys, girls reported more perceived stress when the wording was presented positively, indicating less perceived self‐efficacy to deal with stress. No significant main effects for the three PeSSKi variables were observed for age.

## 7 DISCUSSION

Using a two‐phased design, the Perceived Stress Scale for Kids (PeSSKi), specifically for children aged 7–11 years, was developed and achieved acceptable levels of internal reliability and validity in initial psychometric evaluation. The 10‐item version of the PeSSKi was deemed the most appropriate and psychometrically robust with evidence to support a single factor solution in addition to two subscales differentiated by negatively and positively valenced items. Convergent validity was confirmed through a satisfactory positive association between the PeSSKi (overall score and both negatively and positively valenced factors) and an established measure of worry in children; satisfactory divergent validity was indicated via a negative association with a well known age‐appropriate measure of life satisfaction. Criterion related validity was demonstrated with the PeSSKi being able to discriminate between genders, for the overall PeSSKi score and the positively valenced factor, but not across age groups.

This new scale sought to optimise response reliability using the inclusion of child reporting via appropriate content and delivery of the scale items (Ben‐Arieh, 2005), the use of expertise in generating and adapting the final scale items (Streiner et al., 2015), the inclusion of two social desirability measures (Miller et al., 2015) and the appreciation of common childhood developmental factors in producing an inclusive design (Ozsivadjian et al., 2014). The use of semi‐structured interviews to examine the voice of the children in understanding the linguistic and conceptual properties of stress provided considerable insight in the process of item development, consistent with previous evidence and recommendations (Ben‐Arieh, 2005). The scale aimed to avoid implications of failure or undesirable self‐concepts, having the dual advantage of being agreeable to both the children and the parents and allowing accessibility to children with varying life experience as recommended by previous work (Enlow et al., 2010). This study further demonstrates the capability of the child in being able to successfully respond to a self‐report questionnaire. Consistently, the benefits of involving the voice of children in their own life and healthcare choices is increasingly acknowledged by health professionals (Cremeens et al., 2006). Indeed, potential problems, such as negative wording in some items, appeared not to be a challenge, consistent with other findings for this age‐group (Borgers et al., 2004).

The PeSSKi showed the strongest structure after removal of one item (#10 *There is always someone to talk to about what is bothering me*). This positively valenced reverse scored item was originally included to tap into a feeling of social support, a concept often correlated with perceived stress (Ellis et al., 2009). The ambiguous wording of ‘always’ within the item may have created confusion, possibly challenging literal thinking attributed to this age group, and is consistent with child studies suggesting that vague wording should be avoided to maximise reliability of responses (Borgers et al., 2003). This item may also have overstepped the boundary of global perceived stress by extending too overtly into the domain of social support.

Factor analysis of this perceived stress scale for children was found to parallel that of the well known perceived stress scale for adults (Cohen et al., 1983; Cohen & Williamson, 1988). The factor structure of the 10‐item PeSSKi showed validity in an overall score for perceived stress as well as two factors based on negatively and positively valenced items, consistent with the structure identified by Cohen and Williamson (1988) in their original work and now well established within the adult perceived stress literature utilising the PSS‐10 (e.g. Baik et al., 2019; Barbosa‐Leiker et al., 2013; Golden‐Kreutz et al., 2004; Kechter et al., 2019; Liu et al., 2020; Roberti et al., 2006; Taylor, 2015; Örücü & Demir, 2008). It is particularly interesting that these two factors also emerge in the PeSSKi‐10 child scale of perceived stress and their interpretation fits descriptively with the most frequently used terminology of ‘perceived helplessness’ and ‘perceived self‐efficacy’ applied to the adult PSS‐10 (Roberti et al., 2006). Whilst we would favour the unidimensionality of the PeSSKi to measure perceived stress, given that the two factors identified are based on directionality of items, these two factors are worth future exploration with further child populations including those that differ by culture and clinical status. Consideration of this dual dimensionality of the PeSSKi‐10 in child samples would also contribute to the ongoing debate about the factor structure of the PSS‐10 in the adult perceived stress literature and to the wider debate on the meaning and measurement of stress across the lifespan (Crosswell et al., 2022).

The findings provide initial evidence that the PeSSKi has satisfactory external construct validity in this 7–11 years age group. As hypothesised, the PeSSKi demonstrated convergent validity through a strong positive relationship with generalised worry, whilst retaining a conceptual distinction between the two, as reported in adult populations (Lovibond, 1998; van den Bergh et al., 2021). Evidence for divergent validity was obtained via a weak negative relationship between the SLSS, indicating that children who are more satisfied with their lives are less stressed. This is consistent with young adult (Lee, 2012) and adolescent (Zheng et al., 2019) populations in which a link between perceived stress, self‐efficacy and life satisfaction has been observed, where higher perceived stress is associated with lower feelings of satisfaction.

Findings regarding gender differences in stress perception were also consistent with adult research (Lee, 2012); girls self‐reported significantly more perceived stress than boys. This is in line with work that suggests gender differences in both stress appraisal and response, although previous findings are inconsistent (Hollanders et al., 2017). We found girls had a higher stress score for summed positively worded items of the PeSSKi that made up the perceived self efficacy factor, compared to boys but no gender effects were found for the summed negatively worded items of the PeSSKi constituting the perceived helplessness factor. This gender difference in respect to the positive component of stress perception in this sample of children is partially consistent with previous work using Cohen and Williamson (1988) perceived stress scale (e.g. Taylor, 2015) but in contrast to other studies (e.g. Barbosa‐Leiker et al., 2013; Liu et al., 2020) in adult and adolescent populations. However, it was not the goal of this study to extend these initial findings to examine more complex measurement invariance across gender for specific subscales and further work is needed using the PeSSKi with child populations to adequately address these issues relating to subscales and gender. However, these findings reiterate the importance of incorporating gender awareness into scales and intervention programmes for children even at prepubertal/peri‐pubertal age. Interestingly, in our sample, whilst there were some age differences in response to the PeSSKi, these did not reach significance. This could indicate that either perceived stress across the age range of this sample is broadly similar, or that the scale was capable of interpretation in a developmentally sensitive manner regardless of the age of the child. Either interpretation suggests the applicability of the PeSSKi across the selected age range of 7–11 years.

Despite the promising psychometric properties of the PeSSKi, several methodological limitations from this initial study are acknowledged. Whilst the two scales used to demonstrate validity were appropriate, it may also have been valuable to use an alternative perceived stress scale designed for this age group, such as the PSS‐C (White, 2014). However, we were mindful of not over burdening or confusing our sample through the presentation or repetition of additional scales. Additionally, certain aspects were not assessed in this study. We did not aim to measure sensitivity to change or differentiate between groups already known to exhibit higher stress. Yet initial signs are positive that the scale would be a sensitive measure, as demonstrated by the discriminant validity assessed. It is also acknowledged that cultural differences are present in stress perception and may exert stronger effects than gender (Lee, 2012; Persike & Seiffge‐Krenke, 2016) but cultural variables were not included in the current study. Future work should continue to explore the utility, cogency, and psychometric properties of the measure with different cultural groups to gain validity within different populations. It is encouraging that the parent socio‐economic demographic spread corresponds with latest UK government (NOMIS) figures of occupation status, suggesting that the SES dispersion of the sample was representative. Due to the cross‐sectional nature of the study, future work is needed to examine temporal stability through test‐retest reliability of the scale, sensitivity, and measurement invariance through comparison with known‐groups. Equally, testing at specific points of life transition known to increase stress in children, such as starting school (Turner‐Cobb et al., 2008), could establish normative data and parameters across social milestones. Given that clinical and non‐clinical groups may differ in specificity of responses, assessment of the PeSSKi requires extension across clinical populations and in relation to health outcomes. In keeping with the adult PSS (Cohen et al., 1983) the PeSSKi is not intended for use as a diagnostic instrument. However, convergent validity with the PSWQ‐C suggests that it may be useful in identifying children who could benefit from further support. With regard to sample size we acknowledge that although criteria were met, a larger sample is needed to increase validity particularly in respect to the factor analysis. Finally, the current study relied solely on self‐report and future work is called for to further validate the scale through multiple data sources including physiological measurements including endocrine assessment of cortisol in saliva or hair (Osika et al., 2007).

## 8 CONCLUSION

Overall, these initial findings suggest that the 10‐item PeSSKi offers a robust scale that is easy to administer and straightforward for children to complete. This preliminary study supports the PeSSKi as a reliable and valid measure of perceived stress by attaining the benchmark criteria set out for validating health measures. The current scale is the first global, domain‐free self‐report perceived stress scale designed specifically for 7‐11 year‐old children. In these initial findings the scale shows promise, both conceptually and in acceptability for the target population, enabling self‐report through careful consideration of developmental concerns in linguistic capabilities and response options. It has the potential to support early detection of stress and aid in assessment of intervention effectiveness across a range of populations. Further research is needed to extend these initial findings and determine whether the PeSSKi can demonstrate sensitivity across other non clinical as well as clinical populations, test‐retest reliability in longitudinal work, and its association with health outcomes and objective physiological markers of stress.

## CONFLICT OF INTEREST

The authors declare that they have no conflicts of interest.

## Supporting information

Supporting Information S1

## Data Availability

The quantitative data that support the findings of this study from phase two testing are openly available in Bournemouth University's Online Research Data Repository (BORDaR) at https://doi.org/10.18746/bmth.data.00000209. Qualitative data from phase one of the study in the form of interview transcripts are not shared due to privacy or ethical restrictions.
