# Supplementary material for: The Perceived Stress Scale for Kids (PeSSKi): Initial development of a brief measure for children aged 7–11 years
Source: Stress Health. 2022 Jun 17;39(1):125–36. doi: 10.1002/smi.3174 (PMC10084385; doi:10.1002/smi.3174)
Supplement: Supplementary file 1 — Supporting Information S1 [file SMI-39-125-s001.doc]

**The Perceived Stress Scale for Kids (PeSSKi): Initial development of a brief measure for children aged 7-11 years**

**Supporting Information**

**Contents**

**1 Initial Item Pool for Interview, Issues Identified and Post Interview Revisions Page 2**

**2 Interview Schedule Page 5**

**3 The 11-item PeSSKi with star response scale Page 7**

**4 Item-total statistics for the 11-item PeSSKi Page 10**

**5 Component Matrix (unrotated factor loadings) for PCA of One Factor Solution with 10 PeSSKi items Page 11**

**1 Initial Item Pool for Interview, Issues Identified and Post Interview Revisions**

| Original item # and wording | Issue identified from interview | Post interview revised wording or outcome | Revised 11-Item # |
| --- | --- | --- | --- |
| 1. I get upset when something happens that I don’t expect | Liked but too long | I worry when something unexpected happens | 6 |
| 2. I feel like I am in charge of the important things in my life | Developmental complexity around being in control; ambiguous | Removed | - |
| 3. When something happens that I am not expecting, I feel like I can handle it | Wording too complex | I worry when something unexpected happens | 6 |
| 4. I feel like I have a choice about what is happening to me | Developmental complexity surrounding choice; ambiguous | Removed | - |
| 5. If I have a bad day, I don’t worry because there is always another chance to do better | Universally liked but wording too complex | I feel fine with new things, new people, or new places | 5 |
| 6. Sometimes I get scared that I can’t stop bad things happening | Children did not like to think about bad things and tended to get fixated on them | Removed | - |
| 7. Sometimes I feel like everything gets too much to handle | Sentence too ambiguous | I get upset or angry easily | 8 |
| 8. Sometimes I feel like I am too busy, and I can’t think clearly | Liked but some children did not identify with feeling ‘busy’ | I can think clearly | 1 |
| 9. Sometimes my feelings are so strong, I think I might explode | Liked by most children but younger children took it literally | It is hard to calm down when my feelings are too big or too strong | 9 |
| 10. Sometimes I can’t stop thinking about bad things and I don’t know what to do | Universally liked but too complex | I feel panicky or afraid about little things | 2 |
| 11. When something happens that I don’t expect, I feel scared | The word ‘scared’ was not liked | I worry when something unexpected happens | 6 |
| 12. When I have a problem, I don’t feel like there is anything I can do | Universally liked but too complex | I feel helpless when I have a problem | 4 |
| 13. When somethings bad happens, I know I change it | Children felt they did not have the autonomy to make changes in their lives | Removed | - |
| 14. When something happens that I don’t expect, I feel calm | Good surprises were highlighted and feel excited and happy as well | I feel calm and relaxed | 3 |
| 15. When something bad happens, I know who to ask for help | Knowing who to ask for help was different to feeling able to ask for help | There is always someone to talk to about what is bothering me | 10 |
| 16. When I do something wrong, I feel panicky | Younger children did not like to think about doing things wrong | Removed | - |
| 17. When something new happens, I feel calm | Good surprises were highlighted and how they could feel excited and happy as well. | I feel fine with new things, new people, or new places | 5 |
| 18. When I don’t know what I should be doing, I feel nervous | Liked but conceptually weak, commenting that “nerves are normal” | I worry when something unexpected happens | 6 |
| 19. When I don’t know what is going to happen, I still feel safe | Safety featured as a separate concept to stress | Removed | - |
| 20. I know that I can handle things – even when I don’t understand what is happening | Universally liked but too complex | Whatever happens, I can cope with it | 11 |

**2 Interview Schedule**

Interviewer: *Thanks for agreeing to take part and for answering my questions.*

*Just a reminder that we are audio recording our discussion, but we are not video recording you; also, you have agreed that you will not be recording us during the interview.*

*It will be great to hear what you think about the survey. I don’t want you to answer the questions – I would like to know whether you like them and understand them. There are no right or wrong answers!*

[Selection of PeSSKi items presented to child-parent/guardian dyads at interview – number of questions discussed vary depending on age and timing]

PeSSKi Items

1. I get upset when something happens that I don’t expect
2. I feel like I am in charge of the important things in my life
3. When something happens that I am not expecting, I feel like I can handle it
4. I feel like I have a choice about what happens to me
5. If I have a bad day, I don't worry because there's always another chance to do better
6. Sometimes I get scared that I can't stop bad things happening
7. Sometimes I feel like everything gets too much to handle
8. Sometimes I feel like I am too busy and I can't think clearly
9. Sometimes my feelings are so strong, I think I might explode
10. Sometimes I can't stop thinking about bad things and I don't know what to do
11. When something happens that I don't expect, I feel scared
12. When I have a problem, I don't feel like there is anything I can do
13. When something bad happens, I know I can change it
14. When something happens that I don't expect, I feel calm
15. When something bad happens, I know who to ask for help
16. When I do something wrong, I feel panicky
17. When something new happens, I feel calm
18. When I don't know what I should be doing, I feel nervous
19. When I don't know what is going to happen, I still feel safe
20. I know that I can handle things - even when I don't understand what is happening

[Prompts for child - used in conjunction with discussion of above items as needed]

1. Do you understand the question?
2. Are there any words that are tricky?
3. Do you understand what ‘stress’ is?
4. Do you like how you have to answer the question?
5. Does it make sense to you?
6. Do you think the questionnaire is the right length?
7. Is there anything you think could be better?
8. Can you think of other questions that might be good to use?
9. Is there anything else you would like to say?

[Additional questions and prompts for parent/guardian]

1. What are your thoughts on the questionnaire?
2. Do you think there are any questions that are tricky for your child to understand?
3. Can you think of any improvements?

Any other thoughts?

**3 The 11-item PeSSKi with star response scale**

[For descriptive purposes, not seen by participants]

[Instructions]

Below are some sentences describing how people sometimes think or feel.
Can you look at the sentences and see which ones best describe **YOU**?
Remember – everyone is different!
Some of the sentences might describe you exactly.
Others may not describe you at all.
Other might describe you a just little bit.
For each sentence, please show us in ⭐‘stars’⭐ how it best describes you.

Be honest – this is not a test – there are no right or wrong answers.

[Response scale description and practise]

This is how you can use the stars to tell us your answer.
 The stars will tell us how best a sentence describes **YOU**!
 The stars will tell us how much you feel something.
 If you feel something a lot, fill in all the stars.
 5 means a lot. ⭐⭐⭐⭐⭐
 1 means not at all. ⭐

Now you have a go – show us a lot of stars!

| 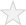 | 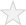 | 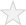 | 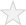 | 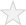 |
| --- | --- | --- | --- | --- |

Now show us not very many stars.

| 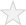 | 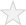 | 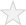 | 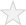 | 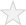 |
| --- | --- | --- | --- | --- |

[Encouragement]

GOOD JOB! 🏆

LET'S GO!


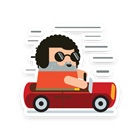


Remember - 5 means a lot. ⭐⭐⭐⭐⭐ 1 means not at all. ⭐ [Regular repeats]

I can think clearly. [PeSSKi item 1]

| 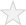 | 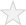 | 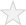 | 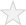 | 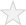 |
| --- | --- | --- | --- | --- |

I feel panicky or afraid about little things. [PeSSKi item 2]

| 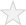 | 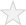 | 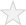 | 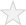 | 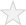 |
| --- | --- | --- | --- | --- |

I feel calm and relaxed. [PeSSKi item 3]

| 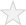 | 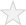 | 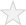 | 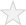 | 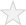 |
| --- | --- | --- | --- | --- |

I feel helpless when I have a problem. [PeSSKi item 4]

| 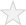 | 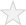 | 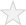 | 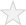 | 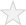 |
| --- | --- | --- | --- | --- |

I like everyone I have met. [Social Desirability item 1]

| 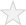 | 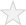 | 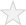 | 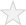 | 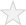 |
| --- | --- | --- | --- | --- |

I feel fine with new things, new people, or new places. [PeSSKi item 5]

| 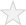 | 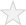 | 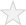 | 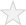 | 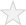 |
| --- | --- | --- | --- | --- |

I worry when something unexpected happens. [PeSSKi item 6]

| 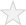 | 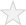 | 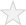 | 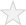 | 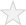 |
| --- | --- | --- | --- | --- |

Remember - 5 means a lot. ⭐⭐⭐⭐⭐ 1 means not at all. ⭐ [Regular repeats]

I sleep well. [PeSSKi item 7]

| 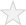 | 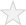 | 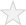 | 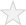 | 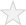 |
| --- | --- | --- | --- | --- |

I always tell the truth [Social Desirability item 2]

| 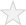 | 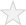 | 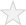 | 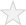 | 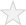 |
| --- | --- | --- | --- | --- |

I get upset or angry easily. [PeSSKi item 8]

| 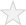 | 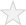 | 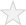 | 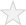 | 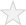 |
| --- | --- | --- | --- | --- |

It is hard to calm down when my feelings are too big or too strong. [PeSSKi item 9]

| 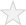 | 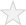 | 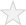 | 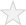 | 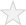 |
| --- | --- | --- | --- | --- |

There is always someone to talk to about what is bothering me. [PeSSKi item 10; Note – this item removed from final scale evaluation due to poor inter-item correlation]

| 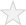 | 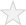 | 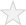 | 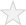 | 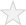 |
| --- | --- | --- | --- | --- |

Whatever happens, I can cope with it. [PeSSKi item 11]

| 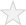 | 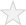 | 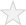 | 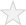 | 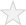 |
| --- | --- | --- | --- | --- |

**4 Item-total statistics for the 11-item PeSSKi**

| **Item #** | **Item wording** | **Scale *M* if item deleted** | **Corrected item-total correlation** | **Cronbach’s α if item deleted** |
| --- | --- | --- | --- | --- |
| 1 | I can think clearly (R) | 25.97 | .45 | .74 |
| 2 | I feel panicky or afraid about little things | 25.32 | .46 | .74 |
| 3 | I feel calm and relaxed (R) | 25.52 | .46 | .74 |
| 4 | I feel helpless when I have a problem | 25.61 | .35 | .75 |
| 5 | I feel fine with new things, new people, or new places (R) | 25.38 | .37 | .75 |
| 6 | I worry when something unexpected happens | 24.87 | .44 | .74 |
| 7 | I sleep well (R) | 25.63 | .50 | .73 |
| 8 | I get upset or angry easily | 24.73 | .43 | .74 |
| 9 | It is hard to calm down when my feelings are too big or too strong | 24.74 | .45 | .74 |
| 10 | There is always someone to talk to about what is bothering me (R) | 26.34 | **.08** | .78 |
| 11 | Whatever happens, I can cope with it (R) | 25.24 | .50 | .73 |

*Note:* Corrected item-total correlations under .3 are indicated in bold. Reverse-scored items are denoted as (R).

**5 Component Matrix (unrotated factor loadings) for PCA of One Factor Solution with 10 PeSSKi items (*N* = 123)**

| **PeSSKi item** | **Component Matrix** | **Communalities** |
| --- | --- | --- |
| 1. I can think clearly | .665 | .381 |
| 2. I feel panicky or afraid about little things | .635 | .392 |
| 3. I feel calm and relaxed | .626 | .403 |
| 4. I feel helpless when I have a problem | .617 | .195 |
| 5. I feel fine with new things, new people, or new places | .615 | .253 |
| 6. I worry when something unexpected happens | .560 | .314 |
| 7. I sleep well | .559 | .443 |
| 8. I get upset or angry easily | .544 | .296 |
| 9. It is hard to calm down when my feelings are too big or too strong | .503 | .312 |
| 11. Whatever happens I can cope with it | .442 | .379 |
